# Autistic young people’s psychological well-being in school

**DOI:** 10.1177/13623613261425010

**Published:** 2026-03-16

**Authors:** Hazel Greer, Caitlin A Williams, Afia Ali, Vaso Totsika

**Affiliations:** 1 University of East London, UK; 2 University College London, UK; 3 University of Birmingham, UK; 4 Queen Mary University of London, UK; 5 East London NHS Foundation Trust, UK; 6 Millennium Institute for Care Research (MICARE), Chile

**Keywords:** academic self-concept, autistic adolescents, school, well-being

## Abstract

Autistic students often face unique challenges at school compared to their non-autistic peers. However, there is a lack of robust evidence investigating their well-being at school. This study examined autistic adolescents’ school well-being using data from a UK population-based cohort. Participants self-reported their positive and negative affect towards and within school. Results indicated that autistic adolescents (*n* = 271; *M*_age_ = 13.73; *SD* = 0.47; 23.11% female) experienced significantly lower levels of school well-being than non-autistic adolescents (*n* = 8077; *M*_age_ = 13.78; *SD* = 0.45; 50.67% female), as evidenced by both lower positive, and higher negative, affect. Differences were attenuated when factors associated with school well-being were controlled for, and levels of well-being no longer differed significantly. Autistic adolescents’ (*n* = 412; *M*_age_ = 13.76; *SD* = 0.46; 18.52% female) positive affect towards school was mostly associated with academic self-concept, whereas negative affect was mostly associated with academic self-concept, bullying victimisation and peer problems. The findings suggest that autistic school well-being could be promoted by supporting autistic students to feel good about their academic effort at school, as well as through school-wide anti-bullying programmes.

**Lay abstract:**

Many autistic young people often experience difficulties at school. However, we know little about how autistic students feel about school compared to their non-autistic peers. Understanding their experiences is important, because school well-being might affect going to school, longer-term learning, as well as friendships and mental health. In this study, we used information from a UK national survey of young people aged 14 years. Students were asked about how happy they felt at school (positive feelings) and how often they felt unhappy at school (negative feelings). We compared answers from autistic and non-autistic students. We also looked at what things were related to autistic students’ feelings about school, including how confident they felt in their schoolwork, experiences of bullying and relationships with friends and family. Autistic students reported feeling less happy and more unhappy at school than non-autistic students. When we considered other things related to well-being, such as being bullying or self-confidence at school, these differences became a lot smaller. For autistic students, positive feelings about school were most strongly related to believing they were good at their schoolwork. Negative feelings about school were related to being bullied, difficult relationships with friends and lower confidence in schoolwork. These findings suggest that differences in school well-being between autistic and non-autistic students may be explained by things that could be changed with support. Supporting autistic students to feel more confident about how well they do academically, and ensuring schools reduce bullying, could help improve autistic young people’s experiences of school.

Well-being is a multi-dimensional construct that brings together two related but distinct philosophical traditions, hedonism and eudaimonia. Hedonic well-being focuses on affect and happiness, and eudaimonic well-being centres on meaning, fulfilment, and other aspects of personal growth (Ryan & Deci, 2001). Well-being impacts on social and emotional development, academic performance, physical health, resilience and health behaviours (Grant et al., 2009; Kubzansky et al., 2018; Sagone & Caroli, 2014), though autistic young people often report lower well-being compared to their non-autistic peers (Featherstone et al., 2024). Given the central role of education in young people’s lives, school contributes substantially to well-being (McGorry et al., 2014; Sawyer et al., 2012; Zhang et al., 2023), and research emphasises the importance of studying schools-specific rather than global well-being (Antaramian et al., 2008). Evidence from research with non-autistic students has suggested school well-being is associated with academic achievement, adaptability, engagement with learning and behaviour within school, as well as long-term functioning and psychological development (Arslan & Coşkun, 2020; Gunawardena et al., 2023; Noble et al., 2008; Noble & McGrath, 2014; Putwain et al., 2019).

Autistic students frequently experience difficulties in the school environment. Research demonstrates that autistic students generally perform less well academically compared to non-autistic students (Baixauli et al., 2021), and are more likely to be absent from school, present with school refusal or be excluded from school compared to their non-autistic peers (John et al., 2022; Munkhaugen et al., 2017; Sasso & Sansour, 2024). These findings suggest that school may be uniquely challenging for autistic students, underscoring the need for research into autistic adolescents’ school well-being (Danker et al., 2016, 2019). While there is emerging evidence that autistic students are not happy at school (National Autistic Society, 2021), there is a lack of robust, population-representative evidence, comparison with non-autistic school well-being levels, and an understanding of factors associated with school well-being among autistic adolescents.

This study aimed to investigate autistic young people’s well-being in school in a population-based sample of autistic 14 year olds. Using population-based data allows for a more representative sample similar to the underlying population compared to data collected using convenience sampling. Well-being is conceptualised through the lens of hedonic well-being (Bradburn, 1969) with a focus on two distinct constructs: positive and negative affect towards and within school (Diener, 1984). These are well-established and measurable indicators of young people’s subjective well-being in school (Ratner et al., 2023; Taylor et al., 2023). Positive affect refers to the presence of positive emotions and feelings towards and within school, while negative affect reflects the presence of negative emotions towards and within school (Diener, 1984; Tian et al., 2013). These indicators are especially relevant for autistic 14 year olds, because early adolescence is a key developmental period in which well-being and school-related well-being often decrease (Casas & González-Carrasco, 2019). In the United Kingdom, this coincides with students beginning their secondary education qualifications and increases in academic pressure (Roome & Soan, 2019), which may be especially challenging for autistic students. In addition, parents tend to report on child well-being differently to the autistic young person themselves (Sheldrick et al., 2012; Stokes et al., 2017), highlighting the importance of self-report for understanding the unique perspective autistic students have on their own internal experience (Keith et al., 2019). The first aim of the study was to investigate levels of autistic young people’s school well-being and whether these are significantly lower compared to their non-autistic peers. Understanding autistic students’ well-being at school offers a route for developing targeted interventions to support autistic students’ experience at school (Boshoff et al., 2025) and likely prevent the well-established inequalities in school attendance and academic outcomes (Baixauli et al., 2021; John et al., 2022; Munkhaugen et al., 2017).

Beyond establishing differences in levels of well-being, research is needed to identify factors, individual or contextual, that are associated with school well-being in autistic students (Li et al., 2024). Well-being at school is likely to be highly contextually influenced. Qualitative evidence has emphasised the importance of relationships with peers and adults, with positive and negative (e.g. bullying) experiences of these relationships being central to school well-being, as well as the role of self-concept and the perception of academic expectations (Boshoff et al., 2025). However, there is a dearth of quantitative studies in autistic samples. Drawing on evidence from research with non-autistic students, factors such as academic performance (Fu et al., 2020; Yang et al., 2019) and self-concept (Rodgers et al., 2018) are likely to be associated with autistic school well-being. Therefore, the second aim of this study was to investigate the association between positive and negative affect in school with individual, family or contextual factors so as to provide an in-depth description of correlates of school well-being that may offer opportunities for targeted support to this highly vulnerable group of students.

## Method

### Sample

This study uses data from wave six of the UK population-based birth cohort study, the Millennium Cohort Study (MCS; University College London, UCL Social Research Institute, Centre for Longitudinal Studies, 2024). The MCS follows individuals born between 2000 and 2002 in Scotland, England, Wales and Northern Ireland. Participants were recruited through the Child Benefits Records which, at the time of recruitment, was a non-means tested benefit with near universal coverage in the United Kingdom. Electoral wards were then randomly selected from benefit records. Recruitment also involved geographically clustering using these electoral wards, ensuring enough participants were recruited from England, Scotland, Wales and Northern Ireland. Stratification was used to over-sample disadvantaged groups from each country. Within England, stratification was also used to over-represent Black and Asian families. Overall, 18,552 families participated in the first MCS wave (MCS1), involving 18,818 children, including target children and their twin and triplet siblings. In the second wave of the MCS (MCS2), another 692 participants were added. All participants gave informed consent. Ethical approval for the MCS was gained through the National Health Service Research Ethics Committee system.

The MCS is ongoing, with several waves of data collected throughout participants’ childhood and adolescence. This study uses data from MCS6 (participants aged 14 years). MCS6 was collected between January 2015 and March 2016. Data regarding autism were drawn from MCS3 (age 5), MCS4 (age 7), MCS5 (age 11) and MCS6 (age 14). A small number of MCS families included information on more than one child (twins or triplets). The first cohort children were randomly selected during recruitment. We included the first cohort children to ensure data remained independent. MCS participants eligible to be included in this study where those for whom there was information available on the presence of an autism diagnosis and who had provided data on at least one of the two study outcomes (positive or negative affect; *N* = 11,347). To compare school well-being between autistic and non-autistic students, analyses were restricted to participants with complete data on all variables (*N* = 8348). Analyses on factors associated with autistic school well-being focused on data from autistic adolescents who had provided information on at least one well-being outcome (positive or negative affect; *N* = 412; see Supporting Information, Figure S1 for flow of participants in MCS).

### Participants

Among the 8348 participants (weighted *M*_age_ = 13.77, *SD* = 0.45; 49.78% male), 271 (3.77%) were autistic and 8077 (96.23%) were not. The demographic profile of the two groups was largely comparable (see Table 1). However, there was a greater proportion of males in the autistic group (*n* = 205, 76.89%) than the non-autistic group (males: *n* = 3951, 50.67%), reflecting the higher rates of autism diagnoses among males. For the within-autism analyses, there were 412 autistic adolescents (*M*_age_ = 13.76, *SD* = 0.46), predominantly male (*n* = 323, 81.48%) and White (*n* = 349, 83.89%) (see Table 1 and Table S1 in the Supporting Information for a comparison of the profile between this group and the 271 autistic adolescents in the between-group analysis).

**Table 1.** Complete-case sample characteristics of autistic and non-autistic young people.

Characteristics	Autistic young people (*n* = 271)	Non-autistic young people (*n* = 8077)
Age (*SD*)	13.73 (*0.47*)	13.78 (*0.45*)
Gender (%)		
Female	66 (23.11%)	4126 (49.33%)
Male	205 (76.89%)	3951 (50.67%)
Ethnicity (%)		
White	233 (86.89%)	6787 (85.51%)
Mixed	14 (4.77%)	378 (5.44%)
Indian	2 (0.37%)	177 (1.58%)
Pakistani	1 (0.18%)	244 (2.05%)
Bangladeshi	1 (0.11%)	99 (0.71%)
Other Asian	2 (0.86%)	45 (0.41%)
Black Caribbean	8 (4.14%)	71 (1.05%)
Black African	4 (1.12%)	127 (1.96%)
Other Black	1 (0.47%)	14 (0.15%)
Chinese	0 (0.00%)	14 (0.18%)
‘Other’ ethnic group	2 (1.10%)	76 (0.97%)
Parent/carer respondent (%)		
Biological parent	270 (99.49%)	8072 (99.94%)
Adoptive parent	1 (0.51%)	4 (0.05%)
Step-parent/partner of parent	0 (0.00%)	1 (0.01%)
Parent/carer respondent gender		
Female	269 (99.65%)	8049 (99.63%)
Male	2 (0.35%)	28 (0.37%)
Socioeconomic status^a^ (*SD*)	4.07 (*1.58*)	4.59 (*1.40*)

Data represent weighted *M* (*SD*), mean (standard deviation); or non-weighted *n/N* (weighted %), non-weighted number of participants (weighted proportion of group).

a Socioeconomic status range: 0–6, greater scores indicating higher socioeconomic status.

### Measures

#### Autism identification

Within MCS3, MCS4, MCS5 and MCS6 when participants were 5, 7, 11 and 14, parents were asked ‘Has a doctor or other health professional ever told you that your child had Autism, Asperger’s Syndrome or other autistic spectrum disorder?’. Any child whose parent said ‘yes’ at any of these time points was identified as being autistic. Parent-report of the presence of a professional autism diagnosis is a valid and reliable method of identifying autistic individuals within research (Daniels et al., 2012; Jagadeesan et al., 2022). Across these waves, 573 (non-weighted) participants were identified as autistic.

#### School well-being

Positive affect was measured using young people’s responses to the question: ‘How do you feel about the school you go to?’ (1 = *completely happy* to 7 = *not at all happy*). For analysis, the rating scale was reversed (0 = *not at all happy* to 6 = *completely happy*), to aid interpretability with higher values showing higher levels of positive affect. Negative affect was measured using young people’s responses to the question: ‘How often do you feel unhappy at school?’ (1 = *all of the time*, 2 = *most of the time*, 3 = *some of the time* and 4 = *never*). As the four points of the original scale did not provide a meaningful distinction between levels of negative affect (e.g. difference between most of the time and some of the time), we combined ‘all of the time’ and ‘most of the time’, creating ‘high negative affect’, and ‘some of the time’ and ‘never’, creating ‘low negative affect’ to aid interpretation of the findings.

#### Correlates of school well-being

Academic self-concept was measured using the academic self-concept grid. This composite measure combines participants’ responses to three items: ‘I am good at maths’; ‘I am good at English’; and ‘I am good at science’, each scored from 0 = *strongly disagree* to 3 = *strongly agree*. The composite academic self-concept measure ranges from 0 to 9, with higher scores indicating a greater academic self-concept.

Bullying victimisation was measured using the question ‘How often do other children hurt you or pick on you on purpose?’ (from 0 = *never* to 5 = *most days*). Similarly, bullying perpetration was measured through asking ‘How often do you hurt or pick on other children on purpose?’ (0 = *never* to 5 = *most days*).

Participants’ closeness with a parent was measured through answers to the question ‘Overall, how close would you say you are to your mother?’ (0 = *not very close* to 3 = *extremely close*). Conflict with a parent was measured through asking ‘How often do you argue with your mother?’ (0 = *never* to 4 = *most days*). Only maternal relationships were considered in the analyses because of the moderate correlation between parents for both closeness and conflict. Consequently, including adolescent reports for both parents would not have provided additional information regarding general parent-child relationships, and might have introduced issues with multi-collinearity. Mother-child relationship was selected for use in the analysis as these variables had slightly stronger associations with school well-being, and more participants had provided data on these variables.

Social support was measured using the Social Support Grid, based on the Social Provisions Scale (Cutrona & Russell, 1987). Three items were included, ‘I have friends and family who help me feel safe, secure and happy’, ‘There is no one I feel close to’ (reverse coded) and ‘There is someone I trust whom I would turn to if I had problems’, each scored from 0 = *not true at all* to 2 = *very true*. We summed these, creating a composite measure. Scores ranged from 0 to 6, with higher numbers indicating greater social support.

A measure of the school social network size was created by combining information from two questions: ‘Do you have any close friends? (friends = other young people)’ (1 = *yes*, 2 = *no*) and ‘How many of your close friends go to the same school as you?’ (1 = *all of them* to 4 = *none of them*). The latter was recoded (0 = *none of them* to 3 = *all of them*), so that greater values indicated more school friends. Those who reported having no close friends or that none of their friends attend the same school as them, were both assigned the value 0. Thus, the created composite variable of social network at school indicates whether they have friends at school from 0 = *none or have no friends* to 3 = *all of them*.

Participants’ peer relationships were measured using the parent-reported peer problems sub-scale of the Strengths and Difficulties Questionnaire (SDQ; Goodman, 1997). This scale comprises five items, each using a 3-point Likert-type-scale (0 = *not true* to 2 = *certainly true*). An example item is my child is ‘rather solitary, tends to play alone’. Scores range from 0 to 10, with higher scores indicating greater peer problems. Internal consistency was adequate within the whole sample (Cronbach’s α = 0.62), and for autistic individuals (Cronbach’s α = 0.72), and was slightly low, though somewhat adequate, for non-autistic individuals (Cronbach’s α = 0.57).

### Analysis

Linear and logistic regression models compared levels of positive and negative affect, respectively, between autistic and non-autistic students. To determine if any differences would still be present after accounting for factors known to be associated with school well-being (identified through prior research), comparisons were repeated accounting for factors known to be associated with school well-being (academic self-concept, bullying victimisation, bullying perpetration, closeness with mother, conflict with mother, social support, social network at school, peer problems and socioeconomic status). Analyses were weighted using population weights to adjust the analytic sample for MCS’ complex sampling design. Non-response weights were applied to adjust for attrition bias. To avoid item-response bias, analyses were conducted using the complete-case sample (*N* = 8348) and a sensitivity analysis (using the full data set) explored the effect of missing data on our findings (presented in results). Analyses were conducted in STATA, in the complex survey environment (svy:).

To investigate factors associated with school well-being among autistic students, multivariable regression models were fitted to look at the association between autistic school well-being and child, school and family factors. Factors potentially associated with school well-being were identified by an initial review of the evidence available from studies involving non-autistic young people (as described in the introduction). The presence of intellectual disability and socioeconomic status (composite measure combining indicators of family poverty, area deprivation, parental unemployment and education, and single-parent status) were also controlled for due to their known association with autism and well-being (Anderson et al., 2025; Maenner et al., 2020; Quon & McGrath, 2014).

To account for the relatively modest sample size of this group (*N* = 412), pairwise correlations were conducted between these and positive and negative affect towards school. Factors associated with these outcomes at *r* ⩾ 0.10 were taken forward to the final regression models. Factors that did not meet this threshold, including intellectual disability, gender, bullying perpetration, happiness regarding friendships and parental involvement with school, were not explored further.

Among the remaining variables eligible to be included in the model, we examined pairwise correlations to investigate potential issues with collinearity. No collinearity was detected, as none of the correlations reached the *r* ⩾ 0.70 threshold. Variables taken forward to the regression models were academic self-concept, bullying victimisation, bullying perpetration, social network at school, peer relationship problems, their closeness and conflict with their mother, social support and family socioeconomic status.

## Results

### Differences in school well-being between autistic and non-autistic adolescents

The unadjusted comparison of positive affect towards school indicated significant, moderate differences between autistic (*M* = 4.03, *SD* = 1.70) and non-autistic individuals (*M* = 4.40, *SD* = 1.52) (*DiM* −0.37 [95% CI −0.63, −0.12], *p* = 0.004) (see Table 2). Similarly, a greater proportion of autistic individuals (*n* = 68, 27.19%) experienced high negative affect within school than did non-autistic individuals (*n* = 1019, 13.54%) (*OR* 2.38 [95% CI 1.67, 3.41], *p* < 0.001). These unadjusted findings indicate that autistic individuals scored 0.37 points lower on positive affect towards school than non-autistic individuals and had 2.38 times greater odds of experiencing high negative affect within school.

**Table 2.** Comparisons between autistic and non-autistic young people’s positive and negative affect within school (weighted and using complete-case sample).

^a^Positive affect towards school	*DiM*	95% CI	*p*
Unadjusted	−0.37	[−0.63, −0.12]	.004
Adjusted for factors likely to be associated with positive affect^b^	0.16	[−0.11, 0.44]	.231
^c^Negative affect within school	*OR*	95% CI	*p*
Unadjusted	2.38	[1.67, 3.41]	<0.001
Adjusted for factors likely to be associated with negative affect^b^	1.01	[0.63, 1.61]	0.964

Autism variable coded as 0 = *non-autistic*, 1 = *autistic*. DiM = difference in means; OR = odds ratio; CI = confidence interval.

a Positive affect towards school was measured on a scale from 0 = *not at all happy* to 6 = *completely happy*.

b Factors likely to be associated with school well-being controlled for in adjusted analyses: academic self-concept, bullying victimisation, bullying perpetration, closeness with mother, conflict with mother, social support, social network at school, peer problems and socioeconomic status. Complete-case sample (unweighted): *N* = 8348, autistic young people: *n* = 271, non-autistic young people: *n* = 8077.

c Negative affect within school was binary (0 = *low negative affect*, 1 = *high negative affect*).

When adjusted to control for factors likely to be associated with school well-being, these differences were reduced. Autistic individuals scored 0.16 points higher on positive affect towards school (*DiM* 0.16 [95% CI −0.11, 0.44], *p* = 0.231) and had equal odds of experiencing negative affect within school (*OR* 1.01 [95% CI 0.63, 1.61], *p* = 0.964).

As part of the sensitivity analysis used to explore the effect of missing data on our findings, weighted unadjusted primary analyses were conducted using the whole data set for comparison. The mean difference in positive affect scores when using the whole data set (*difference in means* [*DiM*] −0.44 [95% CI −0.63, −0.20], *p* < 0.001) was very similar to that from the complete-case sample (*DiM* −0.37 [95% CI −0.63, −0.12], *p* = 0.004) as indicated by overlapping confidence intervals. The odds ratio for negative affect in the whole data set (*OR* 2.26 [95% CI 1.64, 3.13, *p* < 0.001) was almost identical to the odds ration from the complete-case sample (*OR* 2.38 [95% CI 1.67, 3.41], *p* < 0.001). This indicates that missing data had little effect on our findings.

### Factors associated with autistic school well-being

For autistic adolescents (*n* = 412), positive affect towards school was positively associated with academic self-concept (standardised beta regression coefficient [β] 0.18 [95% CI 0.04, 0.33], *p* = 0.013) (see Table 3), meaning a one standard deviation (*SD*) increase in academic self-concept was associated with an increase of 0.16 of a *SD* in positive affect towards school. There were small, non-significant associations with socioeconomic status (β 0.12 [95% CI −0.03, 0.27], *p* = 0.116), bullying perpetration (β −0.07 [95% CI −0.21, 0.07], *p* = 0.319) and bullying victimisation (β −0.08 [95% CI −0.23, 0.06], *p* = 0.248).

**Table 3.** Factors impacting autistic young people’s positive and negative affect towards/within school (weighted).

Predictor variables	Linear regression results (positive affect)			Linear regression results (negative affect)		
	Β	95% CI	*p*	*OR*	95% CI	*p*
^a^Academic self-concept	0.18	[0.04, 0.33]	.013	0.68	[0.58, 0.79]	<0.001
^b^Bullying victimisation	−0.08	[−0.23, 0.06]	.248	1.22	[1.00, 1.48]	.049
^c^Bullying perpetration	−0.07	[−0.21, 0.07]	.319	1.3	[0.92, 1.85]	.140
^d^Social network at school	0.02	[−0.12, 0.16]	.787	1.3	[0.88, 1.93]	.192
^e^Peer problems (SDQ scale)	−0.07	[−0.17, 0.04]	.243	1.19	[1.01, 1.39]	.033
^f^Closeness with mother	0.01	[−0.16, 0.19]	.898	1.13	[0.67, 1.91]	.638
^g^Conflict with mother	−0.05	[−0.2, 0.11]	.558	0.86	[0.59, 1.25]	.419
^h^Social support	−0.02	[−0.18, 0.13]	.754	0.81	[0.6, 1.1]	.177
^i^Socioeconomic status	0.12	[−0.03, 0.27]	.116	0.82	[0.67, 1.01]	.062

β = standardised beta regression coefficient; CI = confidence interval; OR = odds ratio.

a Academic self-concept range: 0–9, with higher numbers indicating greater academic self-concept.

b Bullying victimisation range: 0 = *never* to 5 = *most days.*

c Bullying perpetration range: 0 = *never* to 5 = *most days.*

d Social network at school range: 0 = *none or no friends* to 3 = *all of them* (whether their friends attend the same school as them).

e Peer problems measured using SDQ (Peer Problems Subscale), range: 0–10, with higher scores indicating greater peer problems.

f Closeness with mother range: 0 = *not very close* to 3 = *extremely close*.

g Conflict with mother range: 0 = *never* to 4 = *most days*.

h Social support range: 0–6, higher numbers indicating greater social support.

i Socioeconomic status range: 0–6, higher numbers indicating higher SES.

Negative school affect was associated with autistic academic self-concept (*OR* 0.68 [95% CI 0.58, 0.79], *p* < 0.001), peer problems (*OR* 1.19 [95% CI 1.01, 1.39], *p* = 0.033) and bullying victimisation (*OR* 1.22 [95% CI 1.00, 1.48], *p* = 0.049; Table 3). There were small non-significant associations with bullying perpetration (*OR* 1.30 [95% CI 0.92, 1.85], *p* = 0.140), social network at school (*OR* 1.30 [95% CI 0.88, 1.93], *p* = 0.192) and social support (*OR* 0.81 [95% CI 0.6, 1.1], *p* = 0.177).

## Discussion

To the best of our knowledge, this study is the first to investigate autistic adolescents’ school well-being using quantitative data. As hypothesised, autistic pupils experienced lower levels of well-being in school, reporting lower positive affect and higher negative affect towards and within school. This is an important finding, and it mirrors quantitative research on similar psychological outcomes where autistic young people have been reported to experience lower quality of life, lower psychological well-being levels and greater mental health difficulties compared to their non-autistic peers (Begeer et al., 2017; Lai et al., 2019; Oakley et al., 2021). Current findings, therefore, add to this evidence base, indicating that autistic young people experience lower psychological well-being in school. Interestingly, these differences in school well-being diminished when factors associated with well-being were controlled for, indicating that differences in school well-being might be driven by these factors. This is a promising finding, as it offers a route to understanding and intervening to promote autistic school well-being.

Positive affect towards school was most strongly associated with academic self-concept, whereas negative affect within school was most associated with academic self-concept, peer problems and bullying victimisation. The emergence of academic self-concept as a key correlate of well-being mirrors findings from research on non-autistic young people’s school liking (Ireson & Hallam, 2011) and also findings from qualitative research that links autistic well-being at school to self-identity (Boshoff et al., 2025; Cooper et al., 2024). Qualitative evidence emphasised the importance of self-identity and how autistic young people view themselves in the world of school (Boshoff et al., 2025), while the present findings narrowed the scope of self-identity to academic self-concept and how well autistic students feel they are performing academically. Autistic young people want to do well academically and in the future and enjoy learning (Danker et al., 2019; Jacobs et al., 2021; McNerney et al., 2015) and this desire is impacting their well-being (Boshoff et al., 2025). In our sample of autistic individuals, academic self-concept was associated with both positive and negative affect. Autistic individuals’ academic performances vary greatly (Keen et al., 2016), and autistic students tend to perform less well academically than non-autistic individuals (Baixauli et al., 2021). Consequently, they may have lower academic self-concepts, as academic performance and academic self-concept are associated (Fu et al., 2020). Research suggests the relationship between academic self-concept and performance to be reciprocal (Wu et al., 2021), meaning both areas may need to be considered when improving autistic young people’s school well-being.

Bullying victimisation and peer problems were associated with negative affect within school. This is consistent with research on non-autistic individuals, demonstrating the impact of peer difficulties and bullying victimisation on well-being and psychological well-being in school (Hellfeldt et al., 2019; Przybylski & Bowes, 2017). These findings were expected and consistent with previous research as autistic individuals experience greater bullying victimisation (Maïano et al., 2016), and peer difficulties (Matthews et al., 2020). Positive relationships with peers and staff are central to autistic school well-being (Boshoff et al., 2025; Cooper et al., 2024). Bullying victimisation and peer problems reflect negative social interactions which, while not diminishing positive affect, increase negative affect.

### Strengths and limitations

Our study had various strengths and limitations which should be considered when interpreting these findings. While we drew on population-representative data, we focused on age 14, a time when well-being starts to decline. Findings on well-being may not translate well to younger or older students. In addition, data were collected between January 2015 and March 2016, and responses may not accurately reflect more recent autistic students’ experiences, given significant societal and educational changes. For example, the COVID-19 pandemic had a substantial negative impact on children’s and adolescents’ mental health (Wolf & Schmitz, 2024), and disruptions to social connections and interactions, along with disruptions in education and daily routine, may have disrupted autistic students’ well-being (Branje, 2023; Pellicano & Heyworth, 2025).

The sample was predominantly White and male, limiting the generalisability of the findings to more diverse groups. In addition, the measures available within the MCS data set were not specifically designed for autistic respondents and some items may have been difficult to interpret. Other factors known to affect autistic adolescents school experiences were not measured, such as the school’s ability to meet autistic student’s needs (Brede et al., 2017) and sensory processing differences (Price & Romualdez, 2025). The MCS did not collect data regarding type of school attended (e.g. mainstream or special) and therefore this could not be controlled for in the analysis. School well-being was conceptualised through hedonic well-being. While this is a recognised approach (Bradburn, 1969), our measurement of well-being was constrained by the data available in the MCS.

The descriptive approach (Shmueli, 2010) underpinning the modelling of the second research aim (identifying variables potentially associated with autistic school well-being) was necessitated by the lack of prior evidence. To balance this approach with a modest sample size, a pragmatic decision was made to use pairwise correlations to reduce the initial number of potentially relevant covariates, an approach that is not always considered theoretically helpful (Hafermann et al., 2021). Nonetheless, this study reports on autistic students’ own responses using a population representative sample, to provide important new evidence on correlates of autistic school well-being that future studies, with larger samples, can built on.

### Implications and future research

Findings of lower psychological well-being at school among autistic adolescents compared to peers highlight the need to focus on improving school well-being for this group of students. In our study, after controlling for a range of factors thought to be associated with psychological well-being, group differences attenuated and became non-significant. This suggests that potential risk factors may operate in a similar way between the two groups, though future longitudinal research is needed to investigate likely risk mechanisms.

More crucially, findings offer insight into potential routes for improving autistic school well-being by providing preliminary evidence on factors related to positive and negative school affect. Academic self-concept was associated with both positive and negative affect among autistic adolescents, emphasising the importance of intervention in this area. Evidence suggests that various approaches can improve academic self-concept, including counselling-based, peer feedback, and academic interventions (Elbaum & Vaughn, 2001; Simonsmeier et al., 2020), as well as student-centred approaches to teaching, such as competence-based learning (Kulakow, 2020). Interventions to improve academic self-concept may also improve autistic students’ academic performance as academic self-concept and academic performance have a bi-directional relationship (Fu et al., 2020; Wu et al., 2021). Future research is needed to investigate the effectiveness of such approaches with autistic adolescents, and how best to support autistic students to feel good about their effort and work at school.

Finally, findings highlighted the need to address autistic adolescents’ experiences of bullying victimisation and peer difficulties. School-wide anti-bullying interventions such as the KiVa and the Olweus Bullying Prevention Program have shown promise across different school environments (Garandeau et al., 2023; Ng et al., 2022; Olweus et al., 2019). Research is now needed to establish their effectiveness with autistic students.

## Summary of findings

The study investigated autistic school well-being in school. Autistic 14 year olds seemed to experience lower psychological well-being in school (lower positive affect, higher negative affect) than non-autistic young people though differences were almost fully accounted for by other factors associated with school well-being. Academic self-concept emerged as a key correlate of autistic well-being, being associated with both positive and negative affect. Negative school affect (i.e. being unhappy in school) was additionally associated with peer difficulties and bullying victimisation. As the first study to investigate psychological well-being in a population-representative sample of autistic young people, findings are promising and call for further research into likely risk pathways as well as possible intervention mechanisms.

## Supplemental Material

sj-docx-1-aut-10.1177_13623613261425010 – Supplemental material for Autistic young people’s psychological well-being in school Supplemental material, sj-docx-1-aut-10.1177_13623613261425010 for Autistic young people’s psychological well-being in school by Hazel Greer, Caitlin A Williams, Afia Ali and Vaso Totsika in Autism
