# Supplementary material for: Autistic young people’s psychological well-being in school
Source: Autism. 2026 Mar 16;30(4):1062–72. doi: 10.1177/13623613261425010 (PMC13005895; doi:10.1177/13623613261425010)
Supplement: sj-docx-1-aut-10.1177_13623613261425010 – Supplemental material for Autistic young people’s psychological well-being in school [file sj-docx-1-aut-10.1177_13623613261425010.docx]

**Supplementary Material**

**Figure S1**

*Attrition and Flow of Participants in Millenium Cohort Study (MCS)*

18,552 first cohort children (CM1) participated in MCS1

692 CM1 children added at MCS2

7,518 CM1 children lost between MCS1 and MCS6

11,726 CM1 children took part in MCS6

12 CM1 children removed by MCS due to lost information

11,717 CM1 children with data available for analysis

367 CM1 children did not have data on either well-being outcome

11,347 CM1 children with data on autism status and a well-being outcome at MCS6

2,999 MCS6 children did not have complete data on all exposure, outcome, and confounder variables

10,935 CM1 children excluded as not identified as autistic

(Sample 2) 412 MCS6 CM1 autistic children with data on negative affect within school

(Sample 1) 8,348 MCS6 CM1 children with complete data on all exposure, outcome, and confounder variables

**Table S1**

*Characteristics of Autistic Young People at Age 14 (Weighted)*

| Characteristics | Autistic young people  (*n*= 412) |
| --- | --- |
| **Age** (*SD*) | 13.76 (*0.45*) |
| **Gender (%)** |  |
| Female | 89 (18.52%) |
| Male | 323 (81.48%) |
| **Ethnicity (%)** |  |
| White | 349 (83.89%) |
| Mixed | 19 (5.12%) |
| Indian | 3 (0.29%) |
| Pakistani | 7 (1.32%) |
| Bangladeshi | 3 (0.27%) |
| Other Asian | 2 (0.53%) |
| Black Caribbean | 10 (3.78%) |
| Black African | 6 (3.48%) |
| Other Black | 1 (0.29%) |
| Chinese | 0 (0.00%) |
| Other ethnic group | 4 (1.02%) |
| **Parent/carer respondent (%)** |  |
| Biological parent | 389 (99.28%) |
| Adoptive parent | 1 (0.33%) |
| Step-parent/partner of parent | 0 (0.00%) |
| Grandparent | 1 (0.39%) |
| **Parent/carer respondent gender (%)** |  |
| Female | 384 (98.74%) |
| Male | 7 (1.26%) |
| **Socioeconomic status^a^** (*SD*) | 4.08 (*1.55*) |

*Note.* Data represent weighted *M(SD),* mean (standard deviation); or non-weighted *n/N* (weighted %), non-weighted number of participants (weighted proportion of participants within group).
^a^ Socioeconomic status range: 0-6, greater scores indicating higher socioeconomic status.
